# Comparative Transcriptome Analysis Reveals Molecular Basis Underlying Fast Growth of the Selectively Bred Pacific Oyster, *Crassostrea gigas*

**DOI:** 10.3389/fgene.2019.00610

**Published:** 2019-06-28

**Authors:** Fuqiang Zhang, Boyang Hu, Huiru Fu, Zexin Jiao, Qi Li, Shikai Liu

**Affiliations:** ^1^Key Laboratory of Mariculture, Ministry of Education, and College of Fisheries, Ocean University of China, Qingdao, China; ^2^Laboratory for Marine Fisheries Science and Food Production Processes, Qingdao National Laboratory for Marine Science and Technology, Qingdao, China

**Keywords:** Pacific oyster, RNA-Seq, growth, DEGs, Ka/Ks, alternative splicing

## Abstract

Fast growth is one of the most desired traits for all food animals, which affects the profitability of animal production. The Pacific oyster, *Crassostrea gigas*, is an important aquaculture shellfish around the world with the largest annual production. Growth of the Pacific oyster has been greatly improved by artificial selection breeding, but molecular mechanisms underlying growth remains poorly understood, which limited the molecular integrative breeding of fast growth with other superior traits. In this study, comparative transcriptome analyses between the fast-growing selectively bred Pacific oyster and unselected wild Pacific oysters were conducted by RNA-Seq. A total of 1,303 protein-coding genes differentially expressed between fast-growing oysters and wild controls were identified, of which 888 genes were expressed at higher levels in the fast-growing oysters. Functional analysis of the differentially expressed genes (DEGs) indicated that genes involved in microtubule motor activity and biosynthesis of nucleotides and proteins are potentially important for growth in the oyster. Positive selection analysis of genes at the transcriptome level showed that a significant number of ribosomal protein genes had undergone positive selection during the artificial selection breeding process. These results also indicated the importance of protein biosynthesis and metabolism for the growth of oysters. The alternative splicing (AS) of genes was also compared between the two groups of oysters. A total of 3,230 differential alternative splicing events (DAS) were identified, involved in 1,818 genes. These DAS genes were associated with specific functional pathways related to growth, such as “long-term potentiation,” “salivary secretion,” and “phosphatidylinositol signaling system.” The findings of this study will be valuable resources for future investigation to unravel molecular mechanisms underlying growth regulation in the oyster and other marine invertebrates and to provide solid support for breeding application to integrate fast growth with other superior traits in the Pacific oyster.

## Introduction

Growth is one of the most important traits related to fitness and production for any organism. Traits associated with fast growth have been one of the major breeding goals to enhance the profitability of production for all food animals. For aquaculture species, growth rate is especially important because aquaculture takes place in highly variable water environments, and improvement of growth not only can reduce the input cost but also can decrease the risk of economic loss by shortening culture time. Therefore, genetic breeding of fast-growing varieties or strains have been extensively conducted in aquaculture species.

Growth is a complex trait in marine mollusks, which is genetically controlled but affected by environmental variables such as temperature and food availability (Tamayo et al., 2011; Gosling, 2015). Quantitative genetic analyses revealed that growth rate has a significant genetic component with a mediate-to-high heritability in marine mollusks (e.g., Kong et al., 2015). Growth rate has been positively correlated with the degree of heterozygosity of enzyme-coding genes (Szulkin et al., 2010). This has been shown from a physiological perspective that more heterozygous individuals grow faster and are characterized by higher levels of metabolism and protein turnovers (Bayne and Hawkins, 1997). Identification of basic elements of endocrine and regulatory networks of vertebrates indicated that a similar system could exist in marine mollusks. Growth rate of abalones has been associated with three neuropeptides secreted in neural ganglia (York et al., 2012), supporting the neural control of growth. Many studies in mollusks have also reported the association of genetic mechanisms within insulin-related peptide genes with differences in growth (Kellner-Cousin et al., 1994; Gricourt et al., 2003; Cong et al., 2013; Feng et al., 2014; Alarcon-Matus et al., 2015). Polymorphisms in the genes coding for enzymes responsible for nutrient acquisition, such as amylases and glycogen synthase, have also been identified (Bacca et al., 2005; Prudence et al., 2006). Although many genetic factors associated with growth have been proposed in a number of marine mollusks, critical molecular mechanisms underlying growth remain largely unexplored.

The Pacific oyster (*Crassostrea gigas*) originated in the Pacific Northwest and has been introduced to many countries around the world for aquaculture purposes (Troost, 2010). It is now one of the most widely cultivated shellfish species worldwide, with global production reaching ∼0.6 million tons in 2016 (FAO, 2018). Because of the great value in economics, a number of selective breeding programs based on family and mass selection have been conducted over the years (Langdon et al., 2003; Evans and Langdon, 2006; Dégremont et al., 2010; Li et al., 2011; de Melo et al., 2016). Starting from 2006, we conducted the selective breeding program of the Pacific oyster in China, using the oysters collected from three wild populations in Rushan (China), Miyagi (Japan), and Busan (South Korea). Significant improvement of the growth has been achieved after generations of artificial selection (Li et al., 2011), as reported in other breeding programs (e.g., Langdon et al., 2003; Evans and Langdon, 2006). In 2013, the selectively bred fast-growing oyster strain from our breeding program was certified as an oyster variety by the National Commission for the Examination and Approval of Aquatic Original and Improved Species, Ministry of Agriculture of China.

The fast-growing oyster variety provides us a good research model for studies of growth trait. Physiological energetics analysis of the fast-growing oysters suggested that the selectively bred oysters had a higher energy gain than unselected oysters, while the basal metabolic rate between them was not significantly different. Therefore, fast-growing oysters possess superior energy budget for growth (Zhang et al., 2018). Gene-associated single nucleotide polymorphism (SNP) markers were developed for association analysis of the markers with growth traits, allowing identification of a number of SNP markers with allele frequencies showing a significant difference between fast-growing oysters and unselected commercial control oysters (Wang and Li, 2017). With the fast-growing oysters as the research material, genome-wide analysis of genetic markers and genes would warrant a fine-scale genetic dissection of growth trait in oysters.

Molecular genetic approaches have been rapidly developed in recent years, allowing for identification of genetic markers and genes that are associated with production traits in oysters. Genetic linkage maps of the Pacific oyster have been constructed based on a variety of molecular markers, including amplified fragment length polymorphism (AFLP) markers or combinations of AFLP with microsatellite markers (Li and Guo, 2004; Guo et al., 2012), microsatellites markers (Li et al., 2003; Hubert and Hedgecock, 2004; Hubert et al., 2009; Plough and Hedgecock, 2011), SNPs (Sauvage et al., 2007; Wang et al., 2015; Qi et al., 2017), and a combination of microsatellite markers with SNPs (Sauvage et al., 2010; Zhong et al., 2014; Hedgecock et al., 2015). Based on these linkage maps, quantitative trait locus (QTL) mapping studies have been performed to examine the genetic basis of growth-related traits in the Pacific oyster (Prudence et al., 2006; Hedgecock et al., 2007a; Guo et al., 2012; Wang et al., 2016; Li et al., 2018). In most of these studies, numerous QTLs associated with growth traits were reported, indicating that growth in oysters is a highly polygenic trait (Qin et al., 2012; Gutierrez et al., 2018). However, the identified growth-related QTLs could only explain a limited portion of the phenotypic variation in the Pacific oyster. With more and more studies being conducted, it is being well recognized that integrative analysis of the genetic findings with genomics is required to unravel the molecular mechanisms behind the complex traits such as growth.

The rapidly developed high-throughput sequencing technologies have dramatically boosted genomics research and enabled genetic analysis of traits at whole genome level. RNA-Seq (high-throughput sequencing of RNA) is now an effective tool for transcriptome level analysis of gene expression related to production and performance traits. A large number of RNA-Seq studies have been conducted in the Pacific oyster for analyses of various traits including salinity stress (Zhao et al., 2012; Meng et al., 2013), shell colors (Feng et al., 2015), virus infection (He et al., 2015), heat stress (Yang et al., 2017), and sex determination (Yue et al., 2018). Transcriptome sequencing approach has been applied to understand growth-related traits in the Pacific oyster (Hedgecock et al., 2007b) and other species (e.g., Guan et al., 2017). In the Pacific oyster, the massively parallel signature sequencing (MPSS) was used to generate expressed sequence tags for investigation of genetic causes of heterosis, and it indicated that ribosomal proteins involved in protein metabolism could play a critical role in growth (Hedgecock et al., 2007b). However, low throughput of MPSS technique was not able to provide deep genome coverage data for comprehensive analysis of molecular mechanism underlying growth. In this study, toward understanding of the molecular basis for fast growth in the selectively bred Pacific oyster, we compared the transcriptomes of the selectively bred oysters and unselected wild oysters by a deep RNA-Seq. The transcriptional differences were analyzed from several aspects, including gene expression, gene positive selection, and gene alternative splicing. The results will be valuable for future efforts toward understanding of molecular mechanisms underlying growth regulation and molecular breeding in the Pacific oyster.

## Materials and Methods

### Ethics Statement

The experiments in this study were conducted according to institutional and national guidelines. No endangered or protected species was involved in the experiments of the study. No specific permission was required for the location of the culture experiment.

### Experimental Animals

Samples of the fast-growing oysters used in this study were from the oyster breeding program conducted by our research group (Li et al., 2011). Briefly, the selectively bred line of the Pacific oyster for fast growth was first developed in 2007, using breeding base population constructed with the wild oysters collected from Rushan Bay (36.8°N, 121.6°E), Shandong, China, in 2006. Thereafter, this line was successively selected for fast growth annually and had undergone 10 successive generations of mass selection up to 2017. In June 2017, a total of 110 one-year-old fast-growing Pacific oysters from the selectively bred variety “Haida No. 1” and 120 unselected wild individuals were simultaneously stripped spawned and separately cultured in two 24-m^3^ concrete tanks in a hatchery in Laizhou (37.3°N, 119.9°E), Yantai, China. The larvae were reared according to the procedures as reported in a previous study (Li et al., 2011). The same rearing procedure and feeding were applied to the two tanks. When the spats attached to the collectors (scallop shell) reached 2–3 mm in shell height, they were transferred to Sanggouwan Bay in Rongcheng (37.1°N, 122.5°E, Shandong, China) for marine culture.

### Growth Measurement and Sampling

In December 2017, 100 6-month-old Pacific oysters were collected from each of the two populations (thereafter referred as “breed” and “wild” groups) for use in this study. Shell height, shell length, shell width, and total weight of each individual were measured and weighed. Nine individuals from each of the two groups were randomly chosen for tissue collection. Equal amount of the mantle tissues was dissected from each of three oysters and pooled into one sample, creating three biological replicates for “breed” and “wild” groups, respectively. Tissues were flash frozen in liquid nitrogen and then transferred to −80°C until used for RNA extraction.

### RNA Extraction, Library Construction, and Sequencing

Total RNA was extracted using TRIzol reagent (Invitrogen) according to the manufacturer’s instructions. The RNA quality was confirmed by running 1% agarose gel electrophoresis. RNA concentration and purity were measured using NanoDrop (Thermo Fisher Scientific), and the RNA integrity number (RIN) was assessed using the RNA Nano 6000 Assay Kit of the Bioanalyzer 2100 system (Agilent Technologies).

Six sequencing libraries were constructed using NEBNext^®^ Ultra^™^ RNA Library Prep Kit for Illumina^®^ (NEB, USA) following manufacturer’s protocols. The index codes were added to attribute sequences to each sample. Briefly, mRNA was purified from total RNA using poly-T oligo-attached magnetic beads. First-strand cDNA was synthesized using random hexamer primer and M-MuLV Reverse Transcriptase (Rnase H-). Subsequently, second-strand cDNA was synthesized using DNA polymerase I and RNase H. After purification, end repair, adenylation of 3′ ends of DNA fragments, and adaptor ligation, cDNA fragments of 250–300 bp were selected using AMPure XP beads and enriched by PCR. Library quality was evaluated on the Agilent Bioanalyzer 2100 system. The index-coded samples were clustered on the cBot Cluster Generation System using TruSeq PE Cluster Kit v3-cBot-HS (Illumina) according to the manufacturer’s instructions. After cluster generation, the libraries were sequenced using the Illumina HiSeq 2500 platform for 150-bp paired-end reads.

### Read Mapping and Differential Expression Analysis

Raw reads in fastq format generated from Illumina sequencing were assessed by FastQC. Clean reads were obtained by trimming reads containing adapter, reads containing poly-N, and reads with low sequencing quality. The downstream analyses were based on the high-quality clean reads. The reference oyster genome (Zhang et al., 2012) was first indexed (Li et al., 2009), and then the paired-end clean reads were aligned to the indexed reference genome using Hisat2 (v2.0.4) (Kim et al., 2015). Hisat2 was selected as the mapping tool because it can generate a database of splicing junctions based on the gene model annotation file and thus provide better mapping results than do other non-splicing mapping tools. The counts of reads mapped to each gene were obtained using HTSeq (v0.9.1) (Anders et al., 2014). The fragments per Kilobase of transcript per million mapped reads (FPKM) of each gene was then determined based on the length of the gene and counts of reads mapped to the gene (Trapnell et al., 2010).

Differential expression analysis of the two groups (“breed” vs “wild”) was performed with the R package DESeq (1.18.0) (Anders and Huber, 2010), using a model based on the negative binomial distribution to calculate the *P*-value. The resulting *P*-values were adjusted using the Benjamini and Hochberg approach for controlling the false discovery rate. Genes with an adjusted *P*-value < 0.05 and fold-change > 1.5 were determined as differentially expressed genes (DEGs). Volcano plot was drawn using R scripts to exhibit the overall distribution of DEGs. Gene ontology (GO) enrichment analysis was conducted using the R package GOseq to study the distribution of DEGs in gene ontology in order to clarify the biological meaning as indicated in terms of gene function (Young et al., 2010). GO terms with corrected *P*-value of less than 0.05 were considered as significantly enriched with DEGs. KEGG pathway analysis was conducted to understand high-level functions and utilities of the biological system from molecular-level information (Kanehisa et al., 2007). The statistical enrichment of DEGs in KEGG pathways was tested using KOBAS (2.0) software (Mao et al., 2005), and multiple-testing-corrected *P*-value of less than 0.05 was regarded as significantly enriched in the pathway.

### Quantitative Real-Time PCR Validation

To validate the results of RNA-Seq, 12 differentially expressed genes were selected for quantitative real-time PCR (qRT-PCR) analysis. The RNA samples used for the qRT-PCR assay were same as those used for RNA-Seq. The cDNA was synthesized for qRT-PCR by Prime Script TM RT Reagent Kit with gDNA Eraser (TaKaRa, Dalian, China). Specific primers for qRT-PCR were designed according to the reference sequences using Primer Premier 5.0 (**Supplementary Table 1**). *Eukaryotic elongation factor 1* (*eEF-1*) gene was used as an endogenous control to normalize gene expression by real-time PCR (Renault et al., 2011). The amplification was performed on the LightCycler 480 real-time PCR instrument (Roche Diagnostics, Burgess Hill, UK) using SYBR^®^ Premix Ex Taq^™^ (TaKaRa). Cycling parameters were 95°C for 5 min and then 40 cycles of 95°C for 5 s, 58°C for 30 s, and 72°C for 30 s. The melting curve of PCR products was performed to ensure specific amplification. Relative gene expression levels were calculated by the 2^−ΔΔCt^ method (Schmittgen and Livak, 2008). Data were analyzed by *t*-test using software SPSS 18.0, and *P*-value < 0.05 was considered as statistical significance.

### Alternative Splicing Analysis

Alternative splicing (AS) of genes creates multiple mRNA transcripts from one gene, resulting in tremendous proteomic complexity in higher eukaryotes (Keren et al., 2010; Nilsen and Graveley, 2010). AS events were analyzed using the software rMATS (v3.2.5) (Shen et al., 2014a). The AS events were divided into five categories, including skipped exon (SE), alternative 5′ splice site (A5SS), alternative 3′ splice site (A3SS), mutually exclusive exons (MXE), and retained intron (RI). The expression of each type of AS events was then calculated. The differential alternative splicing (DAS) events were determined from two-group RNA-Seq data with replicates. False discovery rate (FDR) < 0.05 was regarded as the screening criterion for DAS events. Similar to analysis of DEGs, GO and KEGG enrichment analyses were also conducted for the DAS genes.

### Transcriptome *De Novo* Assembly and Annotation

*De novo* assembly of transcriptome was carried out using Trinity (Grabherr et al., 2011) with parameters set as default, followed by mapping cleaned reads to the *de novo* assembled transcript sequences using RSEM software (Li and Dewey, 2011). The assembled transcripts were annotated based on seven public databases, including the NCBI non-redundant protein sequences (Nr) database, NCBI non-redundant nucleotide sequences (Nt) database, Protein family (Pfam) database, euKaryotic Ortholog Groups (KOG) database, Swiss-Prot database, KEGG Ortholog (KO) database, and Gene Ontology (GO) database. For the genes that had multiple assembled transcript sequences, the longest transcript was chosen to represent the gene that is referred to as unigene. Coding sequences (CDSs) were predicted by matching unigenes to the Nr database and Swiss-Prot database by BLASTX.

### Analysis of Positively Selected Genes during Artificial Selection

Putative orthologs between two groups (“breed” vs “wild”) of the Pacific oysters were identified using BLAST-based approach. The CDSs were first extracted from unigenes, and then self-to-self BLASTP was conducted for all amino acid sequences with a cut-off *E*-value of 1E−5, and finally, orthologous pairs were constructed from the BLASTP results with OrthoMCL (v2.0.3) (Li et al., 2003) with default settings. The ratio of the number of nonsynonymous substitutions per nonsynonymous site (Ka) to the number of synonymous substitutions per synonymous site (Ks) was used to test for positive selection. Ka/Ks calculation was performed with PAML (Yang, 2007) package with default settings. The orthologs with Ks > 0.1 were excluded from further analysis to avoid potential paralogs (Elmer et al., 2010). The Ka/Ks ratio greater than 1 usually indicates genes evolving under positive selection (divergent), while those orthologs with a Ka/Ks ratio less than 0.1 indicates that these genes are under heavy selection pressure (conserved).

## Results

### Growth Comparison

Growth of the “breed” and “wild” oysters were compared at 6 months of age as shown in **Table 1**. Apparently, the “breed” oysters showed significant growth advantage to the unselected “wild” oysters in terms of all quantified growth-related traits including shell height, shell length, shell width, and body weight. In addition, the growth-related traits of “breed” oysters were relatively uniformed as indicated by smaller variations of phenotypic traits (**Table 1**).

**Table 1:** Growth comparison between “breed” and “wild” populations of the Pacific oysters.

Populations	Shell height (mm)	Shell length (mm)	Shell width (mm)	Total weight (g)
Breed	71.74 ± 3.5**	32.93 ± 2.7**	18.41 ± 1.7**	20.73 ± 3.3**
Wild	56.44 ± 3.9**	26.17 ± 3.2**	14.93 ± 1.9**	10.99 ± 4.4**

**means the difference is significant at the 0.01 level (P < 0.01). Values are means ± SD, n = 100.

### Transcriptome Sequencing and Mapping

A total of 300.9 million clean reads were obtained after trimming over 307 million 150-bp paired-end raw reads, with Q20 varying from 97.2% to 97.7%. The total bases of clean reads generated from each sample ranged from 7.0 to 8.2 Gb, which is about 15× of the oyster genome size. For the six samples, 79.3–82.2% of the total clean reads were aligned to the genome, of which 71.0–73.1% had a unique alignment and 8.1–9.1% had multiple alignment positions on the genome (**Table 2**). The abundance of transcript sequences for all gene models (35,362) was normalized and calculated by FPKM method using uniquely mapped reads. Nearly half (39.2–47.7%) of the genes were considered not to be expressed or expressed at very low levels (0 < FPKM < 1), and less than 4% (3.4–3.7%) were highly expressed (FPKM > 60). The correlation of gene expression among biological replicates was reasonably high with Pearson’s *R* ^2^ values greater than 0.8 for all samples (**Supplementary Figure 1**).

**Table 2:** Summary of RNA sequencing data and statistics of read mapping to the Pacific oyster genome assembly.

Sample name	DW1	DW2	DW3	ZW1	ZW2	ZW3
Raw reads	48,275,126	53,610,846	55,591,318	47,969,616	51,441,560	50,454,936
Clean reads	47,199,394	52,639,950	54,719,524	46,899,346	50,194,624	49,260,812
Clean bases	7.08G	7.9G	8.21G	7.03G	7.53G	7.39G
Q20 (%)	97.74	97.15	97.29	97.32	97.35	97.36
GC content (%)	43.88	43.27	43.18	43.16	42.80	42.83
Total mapped	38,514,796 (81.6%)	42,635,453 (80.99%)	44,986,750 (82.21%)	37,578,987 (80.13%)	39,807,025 (79.31%)	39,593,155 (80.37%)
Multiple mapped	4,210,112 (8.92%)	4,566,862 (8.68%)	4,983,688 (9.11%)	3,800,378 (8.1%)	4,147,059 (8.26%)	4,119,884 (8.36%)
Uniquely mapped	34,304,684 (72.68%)	38,068,591 (72.32%)	40,003,062 (73.11%)	33,778,609 (72.02%)	35,659,966 (71.04%)	35,473,271 (72.01%)
Exon	88.20%	89.80%	91.40%	89.80%	83.90%	88.80%
Intron	3.00%	3.30%	3.20%	3.40%	3.60%	3.70%
Intergenic	8.80%	6.80%	5.40%	6.80%	12.60%	7.50%

### Analysis of Differentially Expressed Genes

**Figure 1:**
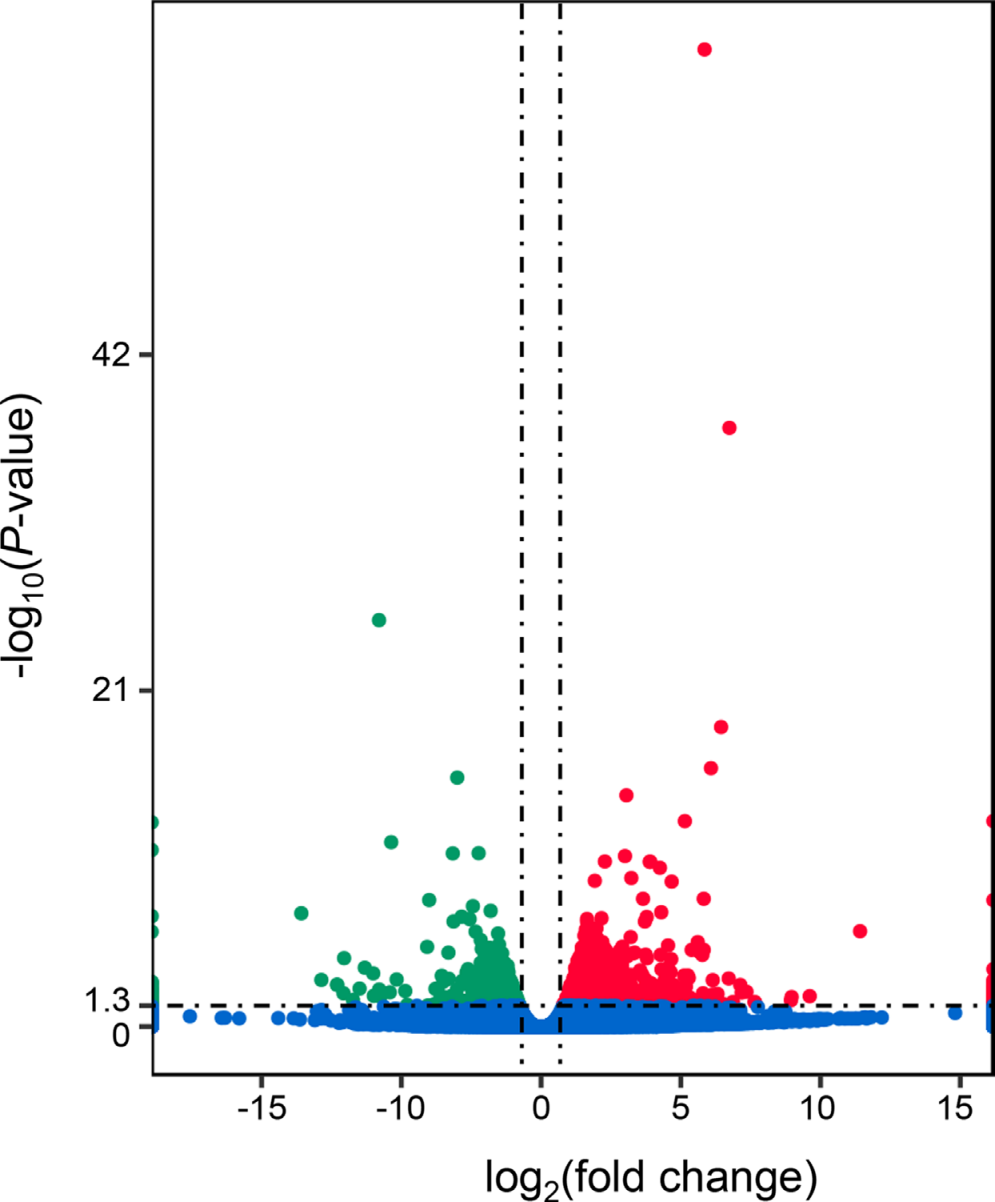
Identification of differentially expressed genes between “breed” and “wild” Pacific oysters. The red dots denote genes that were significantly expressed at higher levels in “breed” oysters, while the green dots denote genes that were significantly expressed at higher levels in “wild” oysters.

A total of 1,303 differentially expressed genes (DEGs) were identified between the “breed” and “wild” Pacific oysters, of which 888 genes were expressed at higher levels in “breed” oysters while 415 genes were expressed at higher levels in the unselected “wild” oysters (**Figure 1** and **Supplementary Table 2**). The number of genes expressed at higher levels in “breed” oysters is significantly larger than that in the “wild” oysters.

**Figure 2:**
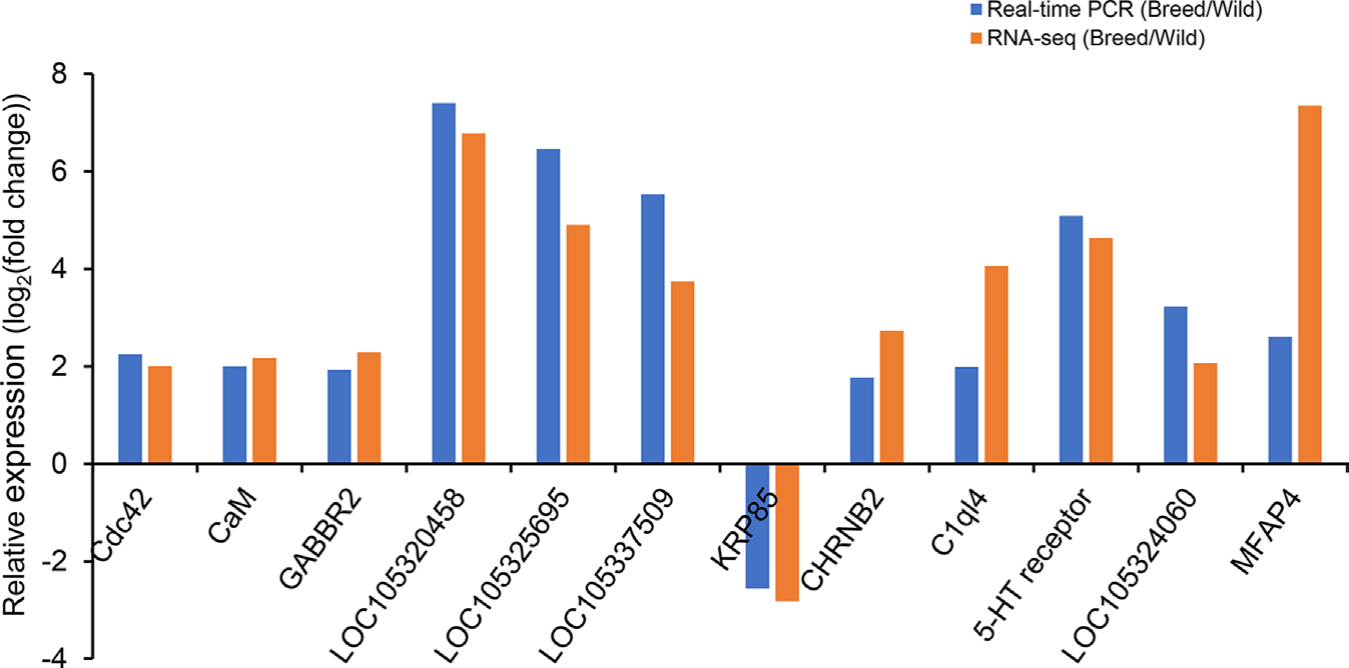
Validation of differentially expressed genes by real-time PCR. *eEF-1* gene was used as internal control.

Twelve DEGs were selected for qRT-PCR validation, and the results were compared with those from RNA-Seq data analysis. The results showed that expression levels of most genes detected by qRT-PCR were consistent with the results as determined based on RNA-Seq analysis, with the exception of *MFAP4*, which showed a similar expression pattern but a significantly different degree of fold change between RNA-Seq and qRT-PCR (**Figure 2**).

**Figure 3:**
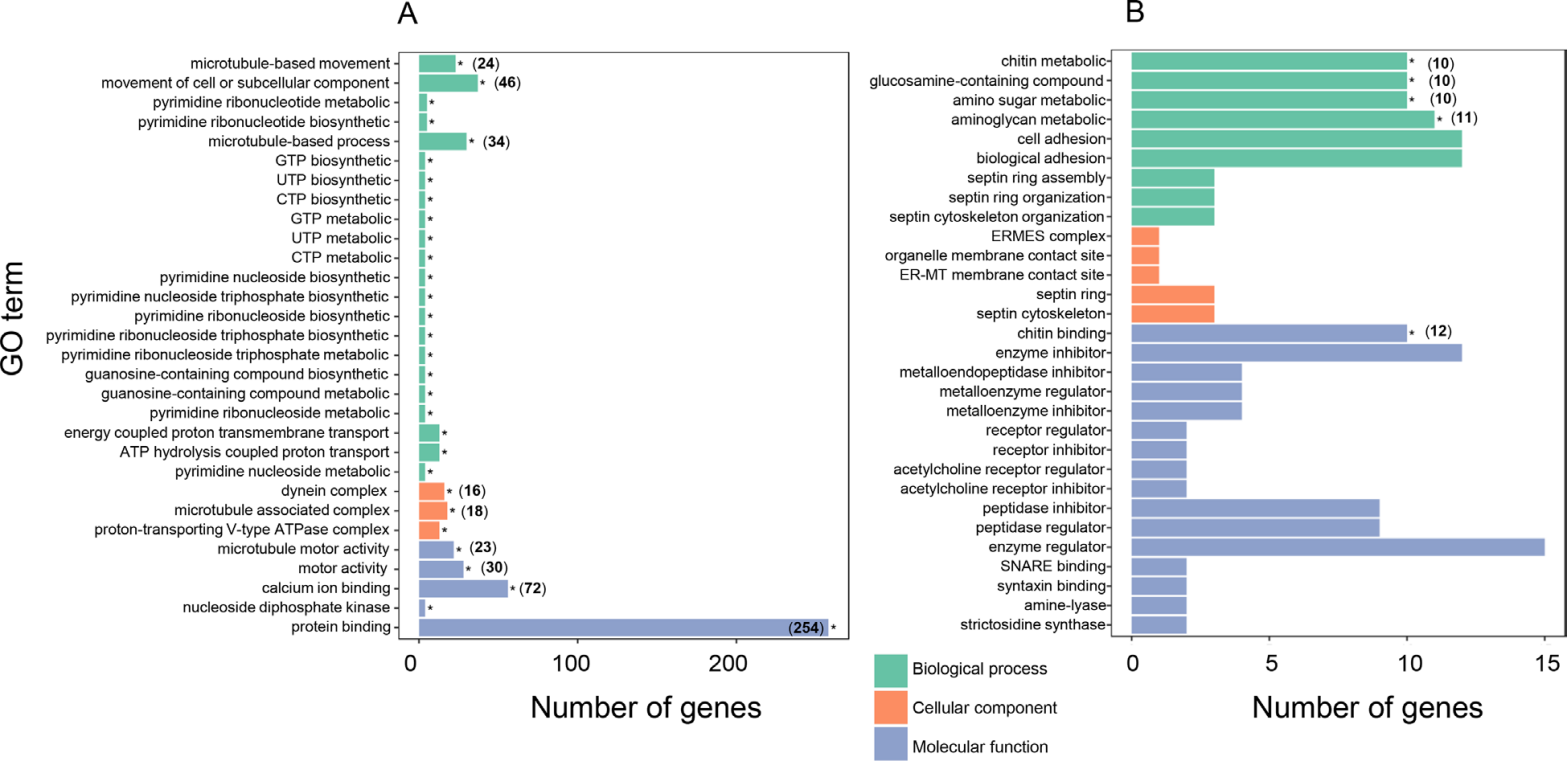
Gene ontology (GO) enrichment analysis of differentially expressed genes between “breed” and “wild” Pacific oysters. **(A)** GO enrichment analysis of differentially expressed genes that were expressed at higher levels in “breed” oysters. **(B)** GO enrichment analysis of differentially expressed genes that were expressed at higher levels in “wild” oysters.

To further understand the biological meanings of these DEGs, gene ontology (GO) term enrichment analysis (*P* ≤ 0.05) was performed. For the 888 genes expressed at higher levels in “breed” oysters, the most significantly enriched GO terms were ”microtubule-based movement” in the biological process (BP), “microtubule motor activity” in the molecular function (MF), and “dynein complex” in the cellular component (CC) (**Figure 3A** and **Supplementary Table 3**). Therefore, microtubule-related genes were highly enriched in top three GO categories in the DEGs expressed at higher levels in the fast-growing “breed” oysters. Besides, significantly enriched GO terms associated with microtubule or cell movement also include “movement of cell or subcellular component,” “microtubule-associated complex,” “motor activity,” and “microtubule-based process” (**Figure 3A**). A total of 42 microtubule-related genes expressed at higher levels in “breed” group were identified. For example, *C1ql4* (LOC105334943) showed 16.7-fold, *DNAH5* (LOC105330782) showed 3.5-fold, and *KIF12* (LOC105329973) displayed a 2.8-fold higher expression in “breed” oyster than “wild” oysters (**Supplementary Table 4**). In addition, genes involved in the process of biosynthesis and metabolism of nucleotide compounds (GTP, UTP, and CTP), ribonucleotide (pyrimidine ribonucleotide), nucleoside triphosphate (pyrimidine nucleoside triphosphate and pyrimidine ribonucleoside triphosphate), and nucleoside (pyrimidine nucleoside and pyrimidine ribonucleoside) were also highly enriched in the DEGs that were expressed at higher levels in the “breed” oysters (**Figure 3A**). A total of four DEGs were involved in these pathways including *NME5* (2.2-fold), *NME7* (2.3-fold), *CiIC3* (2.8-fold), and LOC105346007 (2.4-fold), suggesting that activation of cell movement, microtubule, dynein, and nucleoside compound-related genes may be associated with growth of the Pacific oyster. In addition, a total of 258 DEGs expressed at higher levels in the “breed” oysters were enriched in “protein binding” pathway, with the number of enriched genes far more than that of other pathways (**Figure 3A**).

For the DEGs that were expressed at higher levels in “wild” oysters, the significantly enriched GO terms include “chitin metabolic process,” “glucosamine-containing compound metabolic process,” “chitin binding,” “amino sugar metabolic process,” and “aminoglycan metabolic process” (**Figure 3B**), of which a total of 11 genes were involved, including *Col14a1*, *CHIA*, *EXT*, *ITIH3*, and other seven uncharacterized genes (**Supplementary Table 5**).

**Figure 4:**
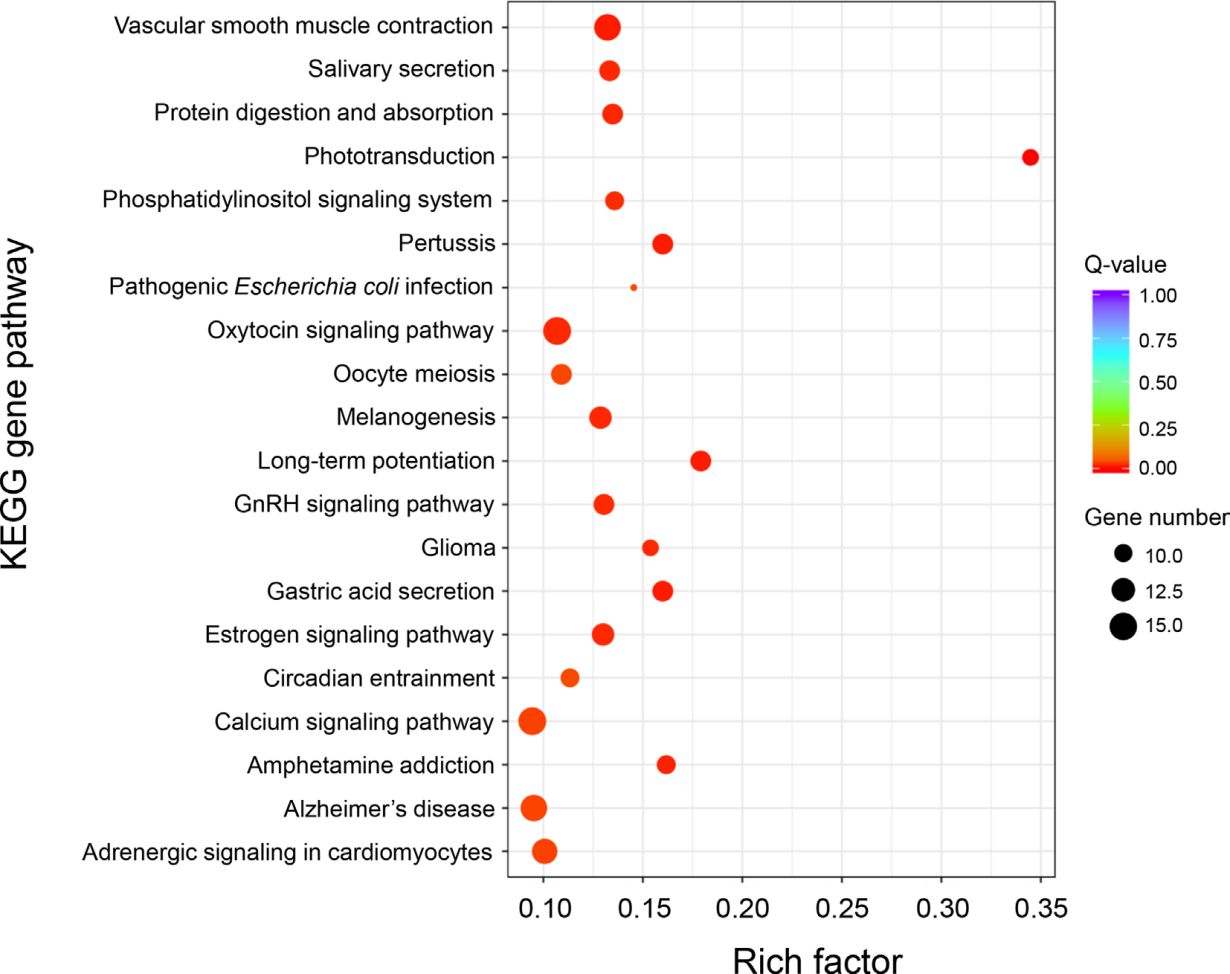
KEGG enrichment analysis of differentially expressed genes between “breed” and “wild” Pacific oysters.

KEGG enrichment analysis of these DEGs was performed to further determine the metabolic processes and signal transduction pathways. The results revealed that the DEGs are significantly enriched in 25 pathways, such as “phototransduction,” “long-term potentiation,” “vascular smooth muscle contraction,” “calcium signaling pathway,” “phosphatidylinositol signaling system,” “gastric acid secretion,” “salivary secretion,” “adrenergic signaling in cardiomyocytes,” and “ABC transporters” (**Figure 4** and **Supplementary Table 6**). A total of 23 genes that had known functions associated with growth regulation were found in these significantly enriched KEGG pathways, and these growth-related DEGs were categorized into different gene families (**Supplementary Table 7**), of which 15 genes were associated with calcium signaling pathway (i.e., *CaM*, *CML*, *CAMK*, *CALCRL*, and *SLC8A*) and two genes were related to actin activity (i.e., *Actin1* and *Actin2*).

### Analysis of Alternative Splicing

A total of 22,573 AS events were identified from 8,176 genes in all six samples, indicating that nearly 24.3% of multi-exonic genes were alternatively spliced. The AS events were categorized into five types, of which ISE and RI events were the most and least frequent, accounting for 75.2% (16,974) and 1.1% (246), respectively (**Table 3** and **Supplementary Figure 2**). The results are consistent with previous studies in animals (Wang et al., 2008), but in contrast to those reported in plants (Marquez et al., 2012; Shen et al., 2014b). To investigate the potential effects of AS on cellular processes related to growth, we identified a total of 3,230 differential alternative splicing (DAS) events from 1,818 genes between “breed” and “wild” oysters. To determine the association of the DAS events with the gene expression, the DAS genes were compared with the DEGs. Only a small subset of DAS genes (175 genes, 9.6%) were differentially expressed between the two groups (**Supplementary Figure 2**).

**Table 3:** Categories of alternative splicing (AS) events in all libraries.

AS events	No. of AS events	Rate of AS events	No. of AS genes	Rate of AS genes*	Average AS per gene
SE	16,974	75.2%	7,346	89.9%	2.3
A5SS	1,156	5.1%	913	11.2%	1.3
A3SS	1,276	5.7%	995	12.2%	1.3
MXE	2,921	12.9%	1,373	16.8%	2.1
RI	246	1.1%	211	2.6%	1.2
Total	22,573	100%	8,176	132.7%	2.8

*The total gene rate is higher than 100% because one gene can experience two or more AS events. Abbreviations: SE, skipped exon; A5SS, alternative 5*′*

splice site; A3SS, alternative 3*′* splice site; MXE, mutually exclusive exons; RI, retained intron.

**Figure 5:**
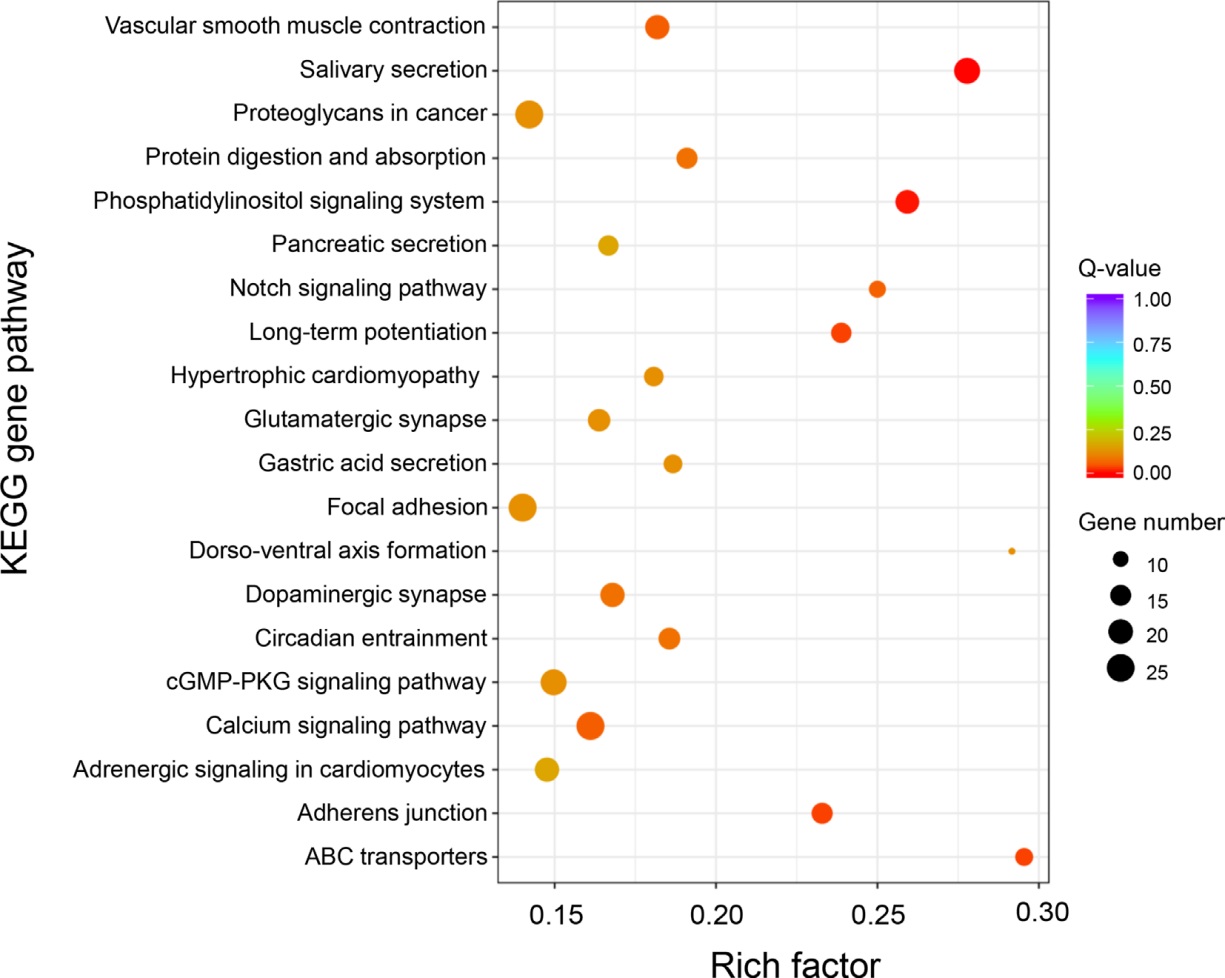
KEGG enrichment analysis of differential alternative splicing genes between “breed” and “wild” Pacific oysters.

Based on GO and KEGG analysis, we found that the DAS genes were significantly enriched in five specific functional pathways, among which “long-term potentiation,” “phosphatidylinositol signaling system,” “salivary secretion,” and “ABC transporters” were also identified as the significantly enriched pathways in DEG analysis (**Figure 5**). A total of 68 genes were involved in these enriched pathways, some of which were from same gene families, such as calmodulin (CaM, three genes), 1-phosphatidylinositol 4,5-bisphosphate phosphodiesterase (PLC, five genes), inositol 1,4,5-trisphosphate receptor (ITPR, four genes), diacylglycerol kinase (DGK, five genes), and ATP-binding cassette (ABC) transporters (13 genes) (**Supplementary Table 8**), among which calmodulins were also identified as DEGs in the differential expression analysis section as mentioned above. Notably, a total of 13 genes that belong to the ABC transporter gene family were enriched in this analysis, indicating the potentially critical roles of the ABC transporters involved in growth of the oysters.

### Identification of Positively Selected Genes During Artificial Selection

In order to identify the positively selected genes during artificial selection of the “breed” oysters, we performed *de novo* transcriptome assemblies with the RNA-Seq data generated for “breed” and “wild” oysters, respectively. This yielded a total of 273,500 transcript sequences for the “breed” oyster group and 241,347 transcript sequences for the “wild” oyster group. By choosing the longest transcript to represent the gene when the genes had multiple transcript sequences assembled, the transcriptome assembly provided 194,978 unigenes for “breed” and 172,863 unigenes for “wild,” respectively (**Supplementary Table 9**). Annotation of the unigenes against the public databases including Nr, Nt, KOG, KO, Swiss-Prot, GO, and Pfam provided a total of 70,419 (36.1%) unigenes from “breed” and 67,173 (38.9%) unigenes from “wild” (**Supplementary Table 10**).

**Figure 6:**
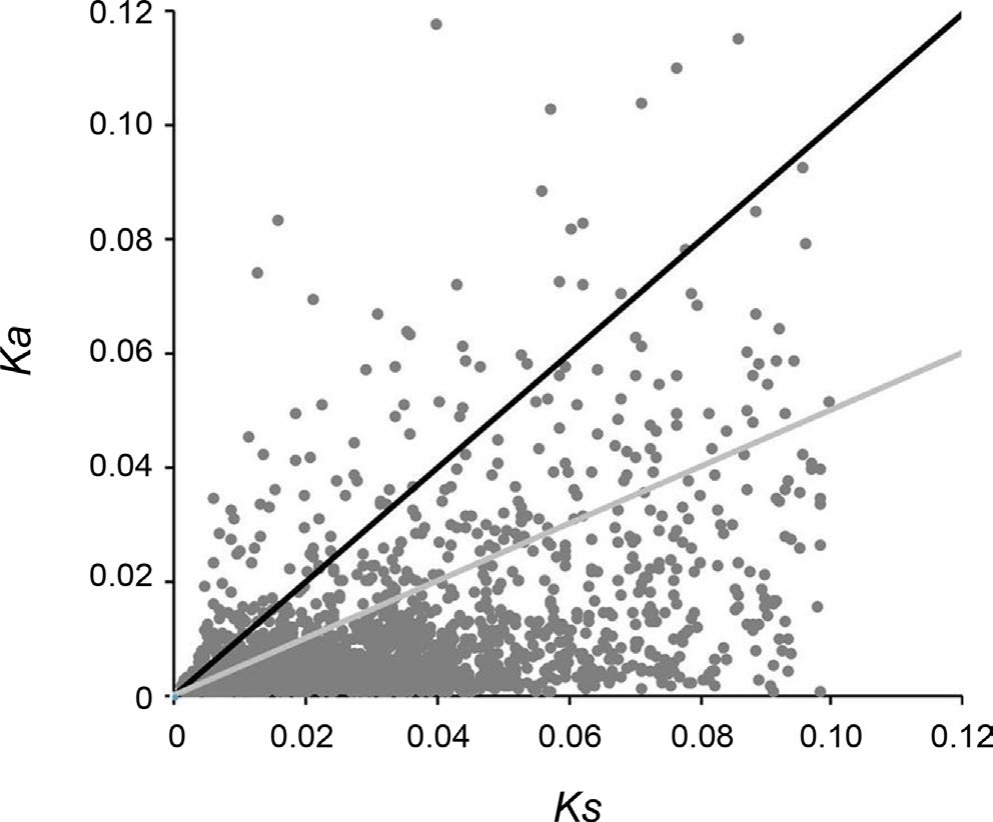
Distribution of Ka/Ks ratio. Black dots above the solid black line indicate orthologous gene pairs identified with Ka/Ks ratio > 1, while the dots between black and gray lines indicate orthologous gene pairs identified with Ka/Ks ratio 0.5–1.

A total of 5,453 pairs of putative orthologs were identified between “breed” and “wild” oysters, of which 3,328 ortholog pairs with all nonsynonymous substitutions and synonymous substitutions were used for calculating Ka/Ks ratios, and the results revealed that 1,198 pairs had Ks > 0.1 that were determined as potential paralogs. After removal of potential paralogs, 2,130 pairs of orthologs were finalized with mean Ka of 0.0097, mean Ks of 0.0304, and mean Ka/Ks ratio of 0.359. A total of 589 ortholog pairs with a Ka/Ks ratio > 1 were identified (**Figure 6**), which might have experienced or be experiencing positive selection during artificial selection.

**Figure 7:**
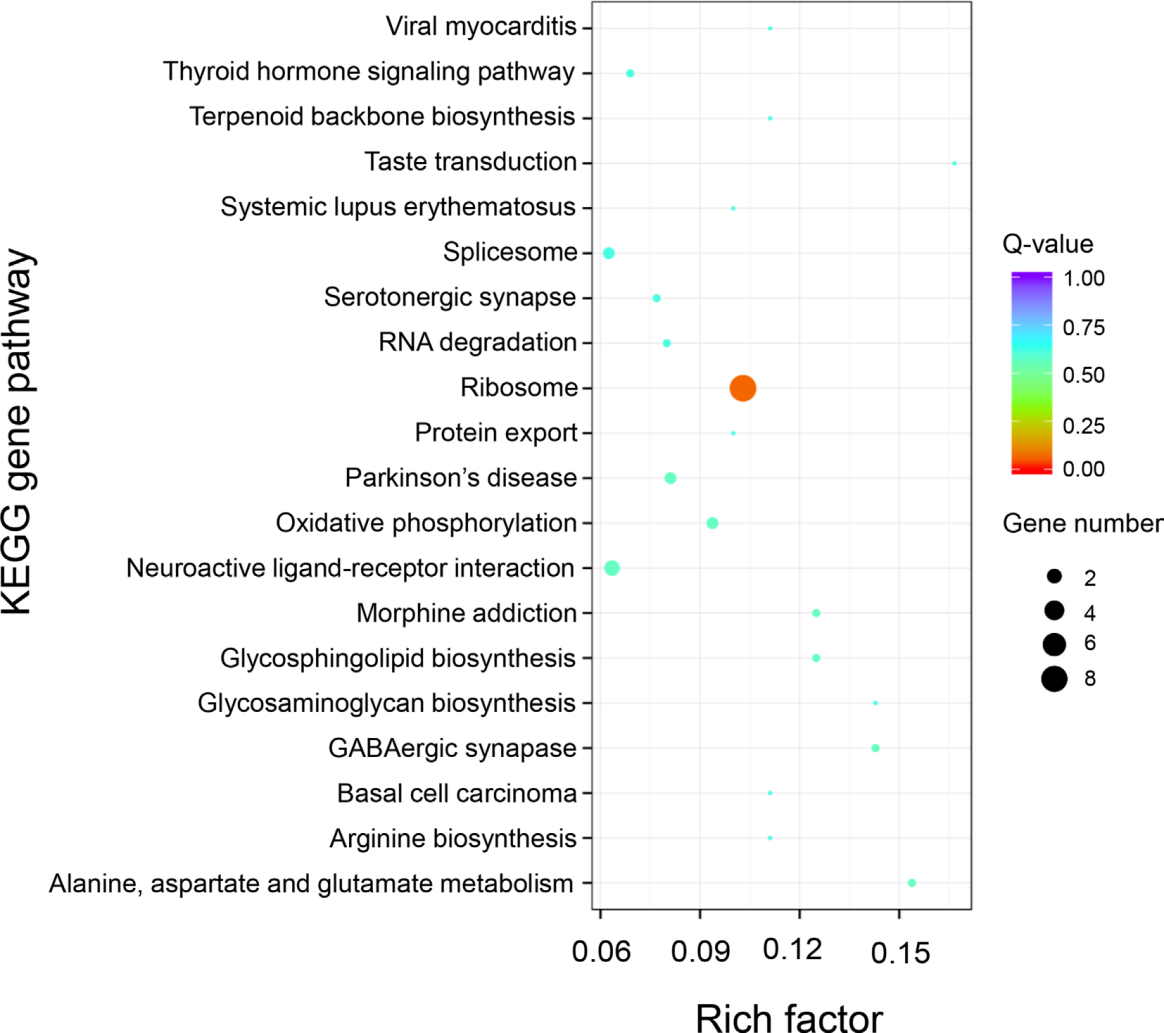
KEGG enrichment analysis of positively selected genes between “breed” and “wild” Pacific oysters.

KEGG pathway analysis of the 589 positively selected genes showed that genes related to ribosomal proteins were greatly divergent between the “breed” and “wild” Pacific oysters. These ribosomal protein-related genes include *RP-L24e*, *RPL24*, *RPS18*, *RP-S3Ae*, *RPS3A*, *RP-L21e*, *RPL21*, *RP-L30*, *MRPL30*, *rpmD*, *RP-L36E*, *RPL36*, *RP-S28e*, and *RPS28* (**Figure 7**).

## Discussion

Growth trait is implicated in a variety of cellular processes and is subject to regulation by multiple complex biological processes. Growth rate is heavily affected by environment variables, especially in aquatic animals inhabiting in highly variable water environments. Generation of fast-growing varieties of animals by selection breeding approach would provide good materials with similar genetic background but contrast phenotype for genetic dissection of growth trait. We initiated a selective breeding program of the Pacific oyster back in 2006. Up to 2017, the selectively bred lines have undergone 10 successive generations of intensive artificial selection for fast growth. Great enhancement of growth has been achieved as indicated by growth trial experiments, while the effects of artificial selection on the Pacific oyster genome remain unexplored. In this study, we used the selectively bred fast-growing oysters as research material to investigate the molecular basis of growth in the Pacific oyster.

We performed transcriptome comparative analysis of the fast-growing selectively bred oysters with the unselected wild oysters. We identified a total of 1,303 protein-coding genes that were differentially expressed (DEGs) between fast-growing oysters and wild controls. Functional analysis of the DEGs showed that microtubule, cell movement, and nucleotide compound-related genes were significantly enhanced for expression in the fast-growing oysters. Microtubules are reported to be essential for proper cell division and cell expansion (Maiato and Sunkel, 2004; Bichet et al., 2008; Jiang et al., 2015). The microtubule-associated proteins (*dynein* and *kinesin*), as well as many other microtubule-related proteins (*C1ql4*, *Cas8*, and *Ift46*), were found to be expressed at higher levels in the selectively bred Pacific oysters. The higher expressions of kinesin genes such as *Kif9* and *Kif12* in the fast-growing oysters are consistent with observations in previous studies that the expressions of *Kif9* and *Kif12* were positively correlated with cell division and cell growth (Gong et al., 2009; Andrieu et al., 2012). The results indicated that microtubule- and cell-movement-related genes could probably play critical roles in growth regulation in the Pacific oysters.

Cell movement plays an important role in the growth and development of organisms, participating in embryonic development and wound healing. The cell movement process needs to be driven by the physical forces generated by cytoskeleton (composed of microfilaments, intermediate filaments, and microtubules) and the participation of many other proteins (Ananthakrishnan and Ehrlicher, 2007). The organization, dynamics, and transport processes of the cytoskeleton are involved in three types of molecular motors, including myosin (which transports cargo along actin filaments) and kinesin and dynein (which transport cargo along microtubules) (Reddy and Day, 2001). As revealed in this study, along with the enhanced expressions of *kinesin* and *dynein* in the fast-growing oyster, the expression of myosin genes, including *Myo3a* and *Myo3b*, was also found to be expressed at higher levels in the fast-growing selectively bred Pacific oysters. Together, the higher expression of cytoskeleton- and cell-movement-related genes in the selectively bred oysters indicated that enhanced division and movement of cells could be probably associated with the fast growth of the Pacific oyster.

The higher expression of genes associated with nucleotide compounds (GTP, UTP, and CTP), pyrimidine ribonucleotide, and nucleoside (pyrimidine nucleoside and pyrimidine ribonucleoside) in the fast-growing oysters suggested the involvement of biosynthesis and metabolism of nucleotides in the growth regulation. Nucleotides carry packets of chemical energy in the form of the nucleoside triphosphates (ATP, GTP, CTP, and UTP) and plays an important role in metabolism at the cellular level, such as synthesis of amino acids and proteins, movement of the cell and cell parts, and division of the cell (Pedley and Benkovic, 2017). The enhanced expression of nucleotide metabolism-related genes, therefore, may contribute to increase the efficiency of protein synthesis and cell division for enhanced growth performance.

Gene functional annotation analysis showed that both differentially expressed genes and alternatively spliced genes were significantly enriched in long-term potentiation, phosphatidylinositol signaling system, ABC transporters, and salivary secretion pathways. In long-term potentiation pathway, differentially expressed genes are mainly calmodulin kinase and its regulators. When long-term potentiation increased, the binding efficiency of Ca^2+^ to calmodulin is increased, causing the increased level of CaMK II and CaMK IV contents. Then, EPK is activated, which promotes increased synthesis of synapse growth protein (Silva et al., 1992; Strack et al., 1997). In this process, calcium ions and calmodulin play critical roles in regulation. Calcium ion carries out its functions by binding to specific calcium receptors or calcium-binding proteins (CaBPs). Genes associated with calcium ion regulation were found to be differentially expressed between “breed” and “wild” groups of the Pacific oysters (**Supplementary Table 7**). For example, the calmodulin-related genes such as *CaM* (LOC105328007), *CML12* (LOC105319978), *CAMK* (LOC105335050), and *SLC8A* (LOC105340116) were expressed 4.5-, 3.2-, 3.0-, and 3.1-fold higher in the “breed” than “wild” oysters, respectively. Exceptionally, the expression of *CALCRL* (LOC105320473) gene was expressed 3.3-fold higher in the unselected “wild” oysters (**Supplementary Table 7**). In the phosphatidylinositol signaling pathway, external signaling molecules bind to G protein-coupled receptors (GPCRs) to activate phospholipase C (PLC), decomposing PIP2 into IP3 and DG and finally activating protein kinase C (PKC) to generate cellular responses, including cell secretion, cell proliferation, and differentiation.

Besides the altered expression patterns of genes between “breed” and “wild” oysters, the effects of artificial selection on these protein coding genes are also of importance. Enrichment analysis of positively selected genes between the “breed” and “wild” groups of the Pacific oysters showed that genes related to the biosynthesis of ribosomal proteins were significantly divergent during the artificial selection process. Ribosomal proteins are crucial for the growth and development of the organisms (Xie et al., 2009; Baloglu et al., 2015). In the larval Pacific oysters, a previous study reported that ribosomal protein-related genes were essentially involved in growth heterosis (Hedgecock et al., 2007b). The divergence of ribosomal protein genes may be associated with differential efficiency of transcription and protein biosynthesis, eventually resulting in growth phenotypic difference between “breed” and “wild” oysters. However, this observation requires future investigation.

## Conclusion

To unravel the molecular basis for fast growth of the selectively bred Pacific oyster, we performed comparative transcriptome analysis of the fast-growing “breed” with the unselected “wild” Pacific oysters in terms of gene expression, alternative splicing, and molecular evolution. The most significant outcome is the identification of potential growth-related genes in the Pacific oysters. Further functional analysis revealed that genes involved in microtubule motor activity, and biosynthesis of nucleotides and proteins would be important for oyster growth. Transcriptome-wide analysis of positively selected genes revealed the important roles of ribosomal protein genes, which further suggested that the process of protein biosynthesis may be a key biological process related to the growth difference between selectively bred oysters and unselected wild oysters. This study provides valuable resources for further investigations on the growth regulation mechanisms and will be useful to support the breeding application to integrate fast growth with other superior traits in the Pacific oysters.

## Data Availability Statement

The Pacific oyster reference genome and gene model annotation files in this study were downloaded from the NCBI (ftp://ftp.ncbi.nlm.gov/genomes/Crassostrea_gigas). All raw RNA-Seq data have been deposited in the NCBI Sequence Read Archive with BioProject accession no. PRJNA524442 (sequence accessions: SRR9089186-SRR9089191).

## Author Contributions

SL conceived and designed the study. FZ, BH, and HF collected the samples and executed the experiments. FZ, BH, HF, ZJ, and SL analyzed the data. FZ drafted the manuscript, and SL revised the manuscript. QL provided reagents and materials and supervised the study. All authors have read and approved the final version of the manuscript.

## Funding

This study was supported by the grants from National Natural Science Foundation of China (31741122 and 31802293), Laboratory for Marine Fisheries Science and Food Production Processes, Qingdao National Laboratory for Marine Science and Technology (2017-2A04), China Postdoctoral Science Foundation (2017M622283), and the Fundamental Research Funds for the Central Universities (201812013).

## Conflict of Interest Statement

The authors declare that the research was conducted in the absence of any commercial or financial relationships that could be construed as a potential conflict of interest.
