# Supplementary material for: Comparative Transcriptome Analysis Reveals Molecular Basis Underlying Fast Growth of the Selectively Bred Pacific Oyster, Crassostrea gigas
Source: Front Genet. 2019 Jun 28;10:610. doi: 10.3389/fgene.2019.00610 (PMC6611504; doi:10.3389/fgene.2019.00610)
Supplement: Supplementary file 1 [file Table_1.docx]

Supplementary Material

## Supplementary Figures

**Supplementary Figure 1**. Correlation analysis of gene expression among biological replicates in the “breed” (ZW1-ZW3) and “wild” (DW1-DW3) Pacific oyster samples.


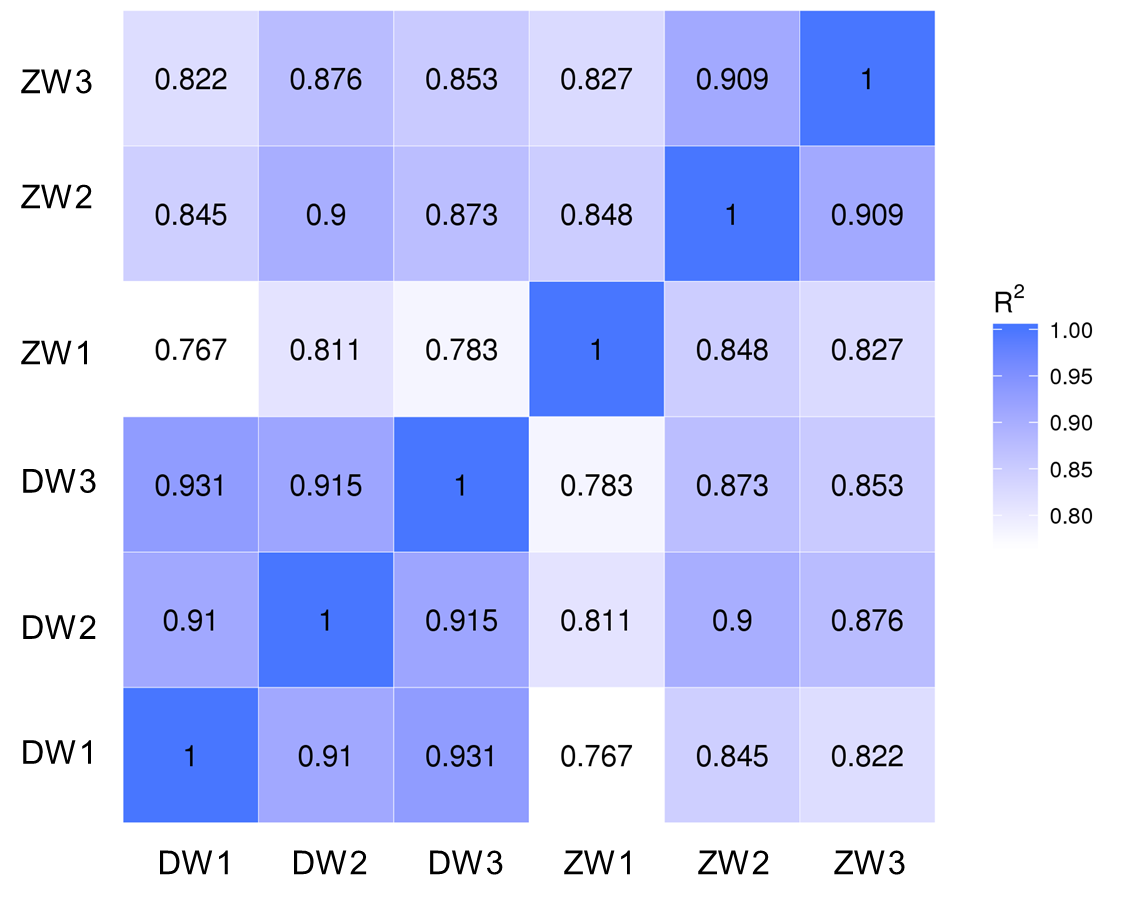


**Supplementary Figure 2**. Distribution of five basic alternative splicing events in all 6 samples. The first column illustrates the intron-exon structure of the AS events, followed by its description, the number of events and percentage.


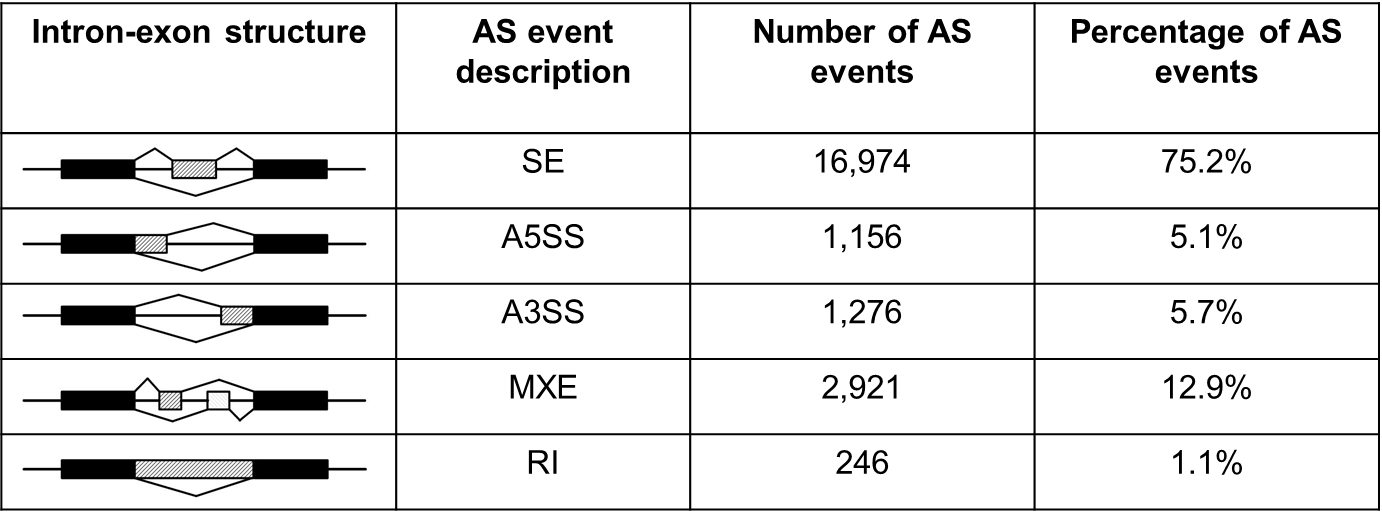


**Supplementary Figure 3**. The comparison of differentially expressed genes and differential alternative splicing genes. DEG for differentially expressed genes and DAS for differential alternative splicing genes.


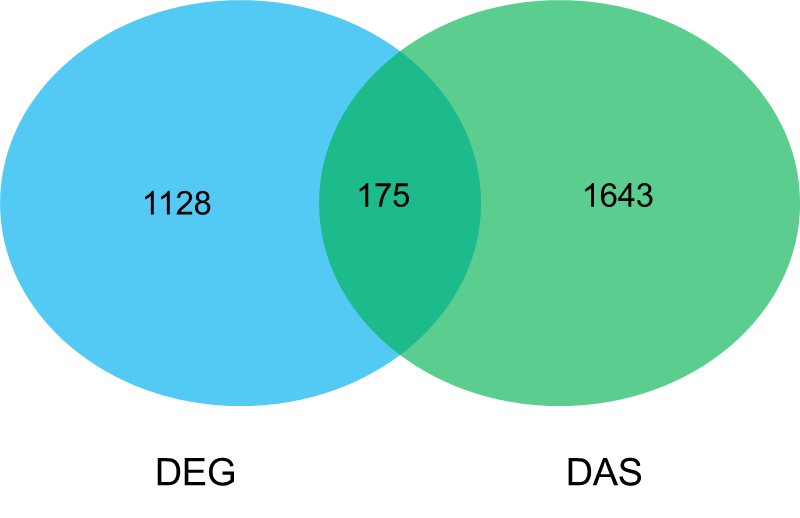


## Supplementary Tables

Supplementary Table 1. Primers used in real-time qPCR for twelve genes

| **Gene ID** | **Gene names** | **Primer (5’ to 3’)** | **Product size (bp)** | **TM (℃)** |
| --- | --- | --- | --- | --- |
| 105328477 | Cdc42 | F: CACCCTGAAGGATGAATACACC  R: GTTCCACGTTATCGAGCGA | 170 | 56 |
| 105328007 | CaM | F: GTCCTCGGTTGGAGAAGAATGT  R: CGTCTACAAACCCATCACAGTCCT | 160 | 58 |
| 105329873 | GABBR2 | F: CGTGGCAGACGAGAAATGTGA  R: AGGTGGGAGCGGATAACAGTAGT | 96 | 58 |
| 105320458 | LOC105320458 | F:TCTCTAAACACAACTCACCAACCTG  R: CAAACAACGACAACGATGCTG | 95 | 58 |
| 105325695 | LOC105325695 | F: AACTGAGTGTGCCTACCAGAATG  R: AAACAAGTGTAGCCATTAGTCCTG | 199 | 58 |
| 105337509 | LOC105337509 | F: CCATCAGACCAACCAGGACC  R: GAGAATGTGTGGATGACCTTGCT | 94 | 58 |
| 105317641 | KRP85 | F:CTGGCACAAATAAACACAACGGA  R: TCCTTCCTCTAAGCGGGTTCC | 120 | 58 |
| 105321010 | CHRNB2 | F: ACTTTAGTGAGCGATAGCATACCCT  R: ATCCTGATGACGAGAACGGTAAG | 122 | 58 |
| 105334943 | C1ql4 | F: CAAGAAGAATGCTCCCGTGG  R: TGGTGAACTTTCCTGTGGTGG | 137 | 58 |
| 105334711 | 5-HT receptor | F: TCTTGTCTTAGGGTTTGCTATTGTG  R: TGCTTATGGATTCCTCGGTCTT | 149 | 58 |
| 105324060 | LOC105324060 | F:CAAGACAAAGAAGGAGGTCATAGAA  R: TCTATGCCATTGCTCTCCTTGT | 169 | 58 |
| 105342748 | MFAP4 | F: TGTAGAGCCTCATTCCACTATCAA  R:AGTCTGTAGTGAGAGGAGAGGGATA | 141 | 60 |

Supplementary Table 2. List of genes that were differentially expressed in the “breed” and “wild” groups of the Pacific oysters.

(See external Excel file)

Supplementary Table 3. Significantly enriched Gene Ontology (GO) terms (P < 0.05) of the genes expressed at higher levels in the “breed” than “wild” groups of the Pacific oysters.

| **Enriched Term(s)** | **Term Type** | **Count** | **FDR** |
| --- | --- | --- | --- |
| microtubule-based movement | Biological process | 23 | 9.77E-09 |
| microtubule motor activity | Molecular function | 22 | 9.77E-09 |
| dynein complex | Cellular component | 16 | 9.77E-09 |
| movement of cell or subcellular component | Biological process | 37 | 4.93E-08 |
| microtubule associated complex | Cellular component | 18 | 4.93E-08 |
| motor activity | Molecular function | 28 | 1.01E-07 |
| calcium ion binding | Molecular function | 56 | 5.34E-06 |
| pyrimidine ribonucleotide metabolic process | Biological process | 5 | 0.002035 |
| pyrimidine ribonucleotide biosynthetic process | Biological process | 5 | 0.002035 |
| microtubule-based process | Biological process | 30 | 0.002035 |
| nucleoside diphosphate kinase activity | Molecular function | 4 | 0.003464 |
| GTP biosynthetic process | Biological process | 4 | 0.003464 |
| UTP biosynthetic process | Biological process | 4 | 0.003464 |
| CTP biosynthetic process | Biological process | 4 | 0.003464 |
| pyrimidine nucleoside triphosphate biosynthetic process | Biological process | 4 | 0.003464 |
| pyrimidine ribonucleoside triphosphate metabolic process | Biological process | 4 | 0.003464 |
| pyrimidine ribonucleoside triphosphate biosynthetic process | Biological process | 4 | 0.003464 |
| CTP metabolic process | Biological process | 4 | 0.003464 |
| GTP metabolic process | Biological process | 4 | 0.003464 |
| UTP metabolic process | Biological process | 4 | 0.003464 |
| pyrimidine ribonucleoside biosynthetic process | Biological process | 4 | 0.003464 |
| pyrimidine nucleoside biosynthetic process | Biological process | 4 | 0.003464 |
| proton-transporting V-type ATPase complex | Cellular component | 13 | 0.010435 |
| guanosine-containing compound metabolic process | Biological process | 4 | 0.012902 |
| guanosine-containing compound biosynthetic process | Biological process | 4 | 0.012902 |
| pyrimidine ribonucleoside metabolic process | Biological process | 4 | 0.015876 |
| energy coupled proton transmembrane transport, against electrochemical gradient | Biological process | 13 | 0.020927 |
| ATP hydrolysis coupled proton transport | Biological process | 13 | 0.020927 |
| pyrimidine nucleoside metabolic process | Biological process | 4 | 0.032202 |
| protein binding | Molecular function | 258 | 0.04296 |
| pyrimidine nucleoside triphosphate metabolic process | Biological process | 4 | 0.043071 |

Supplementary Table 4. List of microtubule-related genes that were differentially expressed in the “breed” and “wild” groups of the Pacific oysters.

(See external Excel file)

Supplementary Table 5. List of differentially expressed genes that were expressed at higher levels in “wild” groups of the Pacific oysters.

| **Gene Family** | **Gene Name(s)** | **Gene Description** | **log_2_(fold change)** | **FDR** |
| --- | --- | --- | --- | --- |
| COL14A1 | LOC105326594 | protein PIF | -1.1642 | 0.0064888 |
|  | LOC105326589 | protein PIF-like | -1.8239 | 0.0062518 |
| CHIA | LOC105332622 | acidic mammalian chitinase | -0.87609 | 0.017269 |
| EXT | LOC105342380 | extensin | -0.85085 | 0.026662 |
| ITIH3 | LOC105336727 | inter-alpha-trypsin inhibitor heavy chain H3 | -1.5026 | 0.00058015 |
| - | LOC105330592 | uncharacterized LOC105330592 | -1.0314 | 0.0024557 |
| - | LOC105345526 | uncharacterized LOC105345526 | -1.4798 | 0.00036403 |
| - | LOC105347916 | uncharacterized LOC105347916 | -0.97753 | 0.0087592 |
| - | LOC105317661 | uncharacterized LOC105317661 | -1.0965 | 0.040583 |
| - | LOC105332574 | uncharacterized LOC105332574 | -1.3659 | 0.046299 |
| - | LOC105337465 | uncharacterized LOC105337465 | -0.86893 | 0.0373 |

Supplementary Table 6. KEGG enrichment analysis of differentially expressed genes between “breed” and “wild” Pacific oysters.

| **Enriched pathways** | **Count** | **P value** | **FDR** |
| --- | --- | --- | --- |
| Phototransduction | 10 | 1.95E-06 | 0.000379 |
| Long-term potentiation | 12 | 5.93E-05 | 0.005777 |
| Vascular smooth muscle contraction | 16 | 0.000103 | 0.005999 |
| Pertussis | 12 | 0.000154 | 0.005999 |
| Gastric acid secretion | 12 | 0.000154 | 0.005999 |
| Amphetamine addiction | 11 | 0.000263 | 0.008562 |
| Estrogen signaling pathway | 13 | 0.000516 | 0.011441 |
| Melanogenesis | 13 | 0.000562 | 0.011441 |
| Oxytocin signaling pathway | 17 | 0.000621 | 0.011441 |
| Protein digestion and absorption | 12 | 0.000625 | 0.011441 |
| Salivary secretion | 12 | 0.000684 | 0.011441 |
| Glioma | 10 | 0.000704 | 0.011441 |
| GnRH signaling pathway | 12 | 0.000814 | 0.012214 |
| Phosphatidylinositol signaling system | 11 | 0.000986 | 0.013731 |
| Calcium signaling pathway | 17 | 0.002142 | 0.026527 |
| Adrenergic signaling in cardiomyocytes | 15 | 0.002177 | 0.026527 |
| Alzheimer's disease | 16 | 0.00264 | 0.030287 |
| Oocyte meiosis | 12 | 0.003216 | 0.033065 |
| Pathogenic Escherichia coli infection | 8 | 0.003222 | 0.033065 |
| Circadian entrainment | 11 | 0.003588 | 0.034313 |
| ABC transporters | 7 | 0.003695 | 0.034313 |
| Inflammatory mediator regulation of TRP channels | 11 | 0.004131 | 0.036618 |
| Rap1 signaling pathway | 18 | 0.004405 | 0.037348 |
| cGMP-PKG signaling pathway | 15 | 0.0058 | 0.047124 |
| Neurotrophin signaling pathway | 12 | 0.006061 | 0.047277 |

Supplementary Table 7. List of growth-related DEGs that were significantly enriched in the KEGG pathways.

| **Gene Family** | **Gene Name(s)** | **Gene Description** | **log_2_(fold change)** | **FDR** |
| --- | --- | --- | --- | --- |
| CaM | LOC105328007 | calmodulin | 2.169 | 0.00137 |
|  | LOC105328002 | calmodulin | 1.8636 | 0.000337 |
|  | LOC105328000 | calmodulin-A | 1.4601 | 0.021831 |
|  | LOC105331896 | calmodulin | -1.188 | 0.000996 |
|  | LOC105332960 | calmodulin-A | 1.5402 | 0.017269 |
|  | LOC105327997 | calmodulin-A-like | 1.7937 | 0.018005 |
|  | LOC105317800 | calmodulin-alpha-like | 1.3283 | 0.016471 |
|  | LOC105317802 | calmodulin-like | 1.5445 | 6.40E-05 |
|  | LOC105327998 | calmodulin-A-like | 1.9768 | 0.014746 |
| CML12 | LOC105319978 | calmodulin-like protein 12 | 1.6663 | 0.018898 |
| CAMK | LOC105335050 | calcium/calmodulin-dependent protein kinase type IV | 1.5796 | 0.026264 |
| CALCRL | LOC105320473 | calcitonin gene-related peptide type 1 receptor | -1.73 | 0.019087 |
|  | LOC105348646 | calcitonin gene-related peptide type 1 receptor-like | -1.4 | 0.026532 |
| SLC8A | LOC105340116 | sodium/calcium exchanger 2 | 1.6173 | 0.004611 |
|  | LOC105319697 | sodium/calcium exchanger 3 | 1.303 | 0.001113 |
| CHRNA | LOC105344576 | neuronal acetylcholine receptor subunit alpha-10 | -1.1588 | 0.010332 |
| CHRNB | LOC105321010 | neuronal acetylcholine receptor subunit beta-2-like | 2.732 | 0.00058 |
| ACTIN | LOC105335713 | actin, adductor muscle | -0.89422 | 0.015415 |
|  | LOC105331444 | actin, alpha skeletal muscle | -1.0401 | 0.031544 |
| PRO | LOC105334064 | profilin-4-like | 1.7497 | 0.010688 |
| MFAP | LOC105342748 | microfibril-associated glycoprotein 4 | 7.3498 | 0.006773 |
| FGFR | LOC105321229 | fibroblast growth factor receptor 4 | 0.84982 | 0.035306 |
| Cdc42 | LOC105328477 | cdc42 homolog | 2.0069 | 0.048842 |

Supplementary Table 8. List of differential alternative splicing genes that were significantly enriched in the KEGG pathways.

(See external Excel file)

Supplementary Table 9. Summary of the transcriptome assembly for “breed” and “wild” groups of the Pacific oyster.

|  | **Number (percentage)** | | | | |
| --- | --- | --- | --- | --- | --- |
|  | **Breed** | |  | **Wild** | |
| **Length (bp)** | **Transcript (bp)** | **Unigenes (bp)** |  | **Transcript (bp)** | **Unigenes (bp)** |
| 200-500 | 168,907(61.76%) | 139,184(71.38%) |  | 149,153(61.80%) | 122,748(71.01%) |
| 500-1000 | 47,766(17.46%) | 29,052(14.90%) |  | 42,186(17.48%) | 25,638(14.83%) |
| 1000-2000 | 32,492(11.88%) | 15,441(7.92%) |  | 28,637(11.87%) | 14,051(8.13%) |
| 2000+ | 24,335(8.90%) | 11,301(5.80%) |  | 21,371(8.85%) | 10,426(6.03%) |
| Count | 273,500 | 194,978 |  | 241,347 | 172,863 |
| Min length | 201 | 201 |  | 201 | 201 |
| Mean length | 778 | 625 |  | 778 | 636 |
| Max length | 28,128 | 28,128 |  | 39,419 | 39,419 |
| N50 length | 1,457 | 1,016 |  | 1,453 | 1,060 |
| Total length | 212,789,269 | 121,949,785 |  | 187,783,274 | 109,977,391 |

Supplementary Table 10. Annotation of Unigenes from the transcriptome assembly for “breed” and “wild” groups of the Pacific oyster.

| **Annotation database** | **“breed” unigenes** | **“wild” unigenes** |
| --- | --- | --- |
| Annotated in Nr | 49,890(25.6%) | 47,556(27.5%) |
| Annotated in Nt | 49,553(25.4%) | 48,677(28.2%) |
| Annotated in KO | 10,086(5.2%) | 10,029(5.8%) |
| Annotated in SwissProt | 20,890(10.7%) | 20,336(11.8%) |
| Annotated in Pfam | 34,122(17.5%) | 31,999(18.5%) |
| Annotated in GO | 34,190(17.5%) | 32,084(18.6%) |
| Annotated in KOG | 10,673(5.5%) | 10,314(6.0%) |
| Annotated in all Databases | 5,189(2.7%) | 5,272(3.0%) |
| Annotated in at least one Database | 70,419(36.1%) | 67,173(38.9%) |
| Total Unigenes | 194,978(100%) | 172,863(100%) |
